# The Admission of Cases of Whooping-Cough

**Published:** 1907-04-20

**Authors:** 


### THE ADMISSION OF CASES OF WHOOPING-COUGH.

What to do with a child who is seriously ill and is suffering from whooping-cough, presents one of the hardest problems with which a house physician or a receiving officer has to deal. To admit it into a ward in which there are other children who are not afflicted with the disease, is to add very considerably to the risks which these run. Whooping-cough is sufficiently serious in itself, but complicating other diseases it becomes a much increased source of danger to child life. At the same time there is grave responsibility in sending back to its squalid and insanitary home a child who is dangerously ill with, say, broncho-pneumonia and whooping-cough. Broncho-pneumonia needs above all things proper nursing and constant medical attention; and the exposure incidental to bringing the child for treatment as an out-patient, usually does more harm than the medicines which are given do good. Drugs are, indeed, the least important part of the treatment of this disease; and yet they form the only possible out-patient measures which can be adopted, for general directions to the out-patient class are largely useless.

Recognising the difficulties and dangers in admitting whooping-cough patients into wards containing children suffering from other diseases, the Great Ormond Street Hospital for Sick Children has wisely set apart a special ward for the treatment of children with this disease. This solves the problem at once. The larger general hospitals might very well follow this example, and might set apart small wards for the reception of cases of pertussis, just as they do for patients with diphtheria. Smaller hospitals, however, being already overburdened, might find this ideal arrangement too expensive. With them we fear the problem for the present will have to remain unsolved.

Even in hospitals which possess special whooping-cough wards, there will always remain the difficulty of recognising the co-existence of this condition in children who have prominent symptoms of other diseases. The parents have a singular habit of suppressing the fact that a child has "whooped," or that it has coughed till it vomited, or that, without definitely doing either of these things, it has for weeks lived in the same room with other children known to be suffering from whooping-cough. They do not as a rule deliberately conceal these facts; they simply do not recognise their significance.

It often happens in the winter months that a child who has long been suffering from whooping-cough, develops broncho-pneumonia, and that, during the progress of the fresh disease, it ceases to "whoop." The unwary house physician, satisfied with the mother's statement that the child has only been ill for a few days, and finding every evidence of dangerous broncho-pneumonia, admits the child forthwith into a general ward, without going further into the history. In the course of the next day or so, the true nature of the case becomes apparent; but by that time other children in the ward may have become infected. This is an accident which does not often happen twice to the same house physician.

It is during convalescence that children with whooping-cough are most liable to develop dangerous broncho-pneumonias. One can thus partly understand how it is that ignorant parents, absorbed in the new and alarming condition, fail to inform the medical officer of a previous disorder, from which, so far as they can tell, the child has already recovered. Nevertheless, a history suggestive of whooping-cough can nearly always be obtained in cases in which this condition has been present. Some day, perhaps, the public will learn to appreciate the grave nature of this disease.

